# Tree Genetic Engineering, Genome Editing and Genomics

**DOI:** 10.3390/ijms232213980

**Published:** 2022-11-12

**Authors:** Matthias Fladung, Birgit Kersten

**Affiliations:** Thünen Institute of Forest Genetics, Sieker Landstraße 2, 22927 Grosshansdorf, Germany

In this Special Issue [1], the focus is on recent research and developments in the use of biotechnological techniques such as genetic engineering, genome editing and genomics to improve tree species and to deepen our understanding of the genetic basis of important tree-specific traits. This is, under the light of ongoing climate change, it is particularly necessary to ensure the availability of stable forest ecosystems with healthy trees in the future. Healthy trees are also important in meeting the growing demand for renewable wood resources. Unfortunately, genetic improvements in tree species by conventional breeding are challenging because of the extended vegetative phases of trees in reaching maturity and because of their individual longevity. Molecular and biotechnological methods offer numerous possibilities for tree improvement, potentially greater than for agricultural crops.

Since the first report on genetic engineering in 1987 in poplar [2], both genome editing [3] and genomics of poplar and various forest, fruit and ornamental tree species have accelerated in an unimaginable manner. Today, the amount of protocols for genetic engineering and genome editing of many tree species is numerous. For a wide range of tree species, huge genomic resources have been built-up, ready-to-use for their application in tree improvement programs. 

In this Special Issue, eight papers dealing with special topics of genomics and biotechnology in different tree species are published. All papers underwent a strict peer-review process to ensure high scientific value. The eight contributions comprise two review paper and six original research papers. The species considered are apple, silver birch, poplar and blackberry/raspberry, and the topics comprise the use of the CRISPR/Cas12a system in different tree species, e.g., to knockout lignin and cellulose synthesis biosynthesis genes in poplars, as well as genomic and functional genomic studies, such as a genome-wide analysis of an Aquaporin gene family.

The review paper entitled “From Genome Sequencing to CRISPR-Based Genome Editing for Climate-Resilient Forest Trees” by Hieu Xuan Cao and colleagues summarizes and discusses the latest progress, opportunities and challenges of genome sequencing and CRISPR-based technology for improving the sustainability of forest tree species. Rapid, efficient and low-cost genome sequencing tools are important in identifying numerous genes and biological processes that are associated with important traits such as wood quality, drought or pest resistance. With this knowledge, the selection of suitable genes as genome editing targets is feasible.

The second review “Achievements and Challenges of Genomics-Assisted Breeding in Forest Trees: From Marker-Assisted Selection to Genome Editing” by Sunny Ahmar et al. describes genomic selection methods such as genome-wide association studies (GWASs) and genomic selection (GS) for various forest tree species. Assisted rapid molecular breeding of trees in combination with advanced biotechnological techniques such as CRISPR/Cas9 is feasible in principle. However, unfortunately, many forest tree species still lack an efficient transformation method and/or an inefficient number of genotypes are accessible for genome editing. A solution for this problem could be the newly developing techniques using growth-regulating factors (GRFs) alone or combined with GRF-interacting factors (GIF). 

Genome editing technology based on CRISPR/Cas9 is commonly applied for different tree species. However, the use of the CRISPR/Cas12a system in apple is described for the first time in “Tracing CRISPR/Cas12a Mediated Genome Editing Events in Apple Using High-Throughput Genotyping by PCR Capillary Gel Electrophoresis” by Susan Schröpfer and Henryk Flachowsky. To prove the functionality of CRISPR/Cas12a in apple, guide RNAs targeting different exons of the endogenous reporter gene *MdPDS*, whose inactivation results in the albino phenotype, were designed. Following transformation, knockout of *MdPDS* could be confirmed by fluorescent PCR capillary electrophoresis and amplicon deep sequencing.

Three papers deal with the model tree genus *Populus* and cover important issues such as cambial activity, lignin content and cellulose biosynthesis. The paper by Michael Riefler and co-workers entitled “A Constitutively Active Cytokinin Receptor Variant Increases Cambial Activity and Stem Growth in Poplar” describes experiments to genetically engineer cambial activity in poplar by expressing the cytokinin biosynthesis gene *ROCK4* or *ROCK3*, a gene encoding a constitutively active cytokinin receptor variant. Both genes were either under the control of their own promoter or the cambium-specific pHB8 promoter. While *ROCK4*-transgenic plants were revealed to be smaller with more lateral branches than the wildtype, *ROCK3*-transgenic plants formed more cambial cells, resulting in an increased growth.

The second poplar-paper “CRISPR-Knockout of CSE Gene Improves Saccharification Efficiency by Reducing Lignin Content in Hybrid Poplar” by Hyun-A Jang et al. deals with the CRISPR/Cas9-based knockout of the lignin gene CAFFEOYL SHIKIMATE ESTERASE (CSE). Poplar plants without functional CSE genes revealed significantly reduced lignin content and higher saccharification efficiency with irregularly shaped xylem vessels but were morphologically indistinguishable from wildtype plants. These characteristics remained stable in a field test covering four seasons.

A CRISPR/Cas9-based knockout approach was also followed to study the biological function of two *SITOSTEROL GLYCOSYLTRANSFERASE* (*SGT*) genes involved in cellulose biosynthesis. Yinxuan Xue and colleagues show in their paper “Investigation of *PtSGT1* and *PtSGT4* Function in Cellulose Biosynthesis in *Populus tomentosa* Using CRISPR/Cas9 Technology” that sucrose and fructose were significantly downregulated in the stems and leaves of mutant plants; however, the glucose levels did not change significantly in roots and stems of *PtSGT1* mutants. The results obtained deepen our understanding of cellulose synthesis in the cell walls of woody plants.

Aquaporins are transmembrane proteins forming so-called Aquaporin water channels (AQPs) that play an important role in water flux across membranes. In silver birch, the diversity, protein features and biological functions are still unknown. Jean-Stéphane Venisse et al. presents in “Genome-Wide Identification, Structure Characterization, and Expression Pattern Profiling of the Aquaporin Gene Family in *Betula pendula*” the first detailed genome-wide analysis of the *AQP* gene family in a birch species. The authors contribute to a better understanding of the specific functions of the *BpeAQP* genes in the responses of silver birch trees to cold stress.

Finally, the problem of lacking appropriate reference genes for RT-qPCR studies in different species of blackberry and raspberry (*Rubus* spec.) was considered in the methodical study “Selection and Validation of Candidate Reference Genes for Gene Expression Analysis by RT-qPCR in *Rubus*” by Yaqiong Wu et al. The authors selected representative cultivars of blackberry and raspberry for RT-qPCR combined with different internal stability analysis software programs and identified the two most stable genes (*RuEEF1A* and *Ru18S*), which can be used as reference genes for future studies of the analysis of differential gene expression in blackberry and raspberry.

All papers published in this Special Issue are significant contributions to tree genetic engineering, genome editing and genomics. Although not all applications are commercialized yet, the editors believe that all approaches described improve our knowledge in these scientific fields, especially in understanding the genetic basis of traits relevant to climate change in trees and the biological function of their underlying genes. These approaches are also worth exploring in future practical applications to preserve forests amidst ongoing climate change.
